# CFD Modelling and Optimization Procedure of an Adhesive System for a Modular Climbing Robot

**DOI:** 10.3390/s21041117

**Published:** 2021-02-05

**Authors:** Miguel Hernando, Virgilio Gómez, Alberto Brunete, Ernesto Gambao

**Affiliations:** 1 Centre for Automation and Robotics (UPM-CSIC), Universidad Politécnica de Madrid, 28012 Madrid, Spain; alberto.brunete@upm.es (A.B.); ernesto.gambao@upm.es (E.G.); 2 Escuela Técnica Superior de Ingeniería y Diseño Industrial, Universidad Politécnica de Madrid, 28012 Madrid, Spain; virgilio.gomez.lambo@alumnos.upm.es

**Keywords:** modular climbing robots, vacuum generation, radial impeller, computer fluid dynamics, test rig measurements, optimization

## Abstract

Adhesion systems are very important in robots for infrastructure inspection (especially in vertical walls). They present the challenge of optimizing the ratio vacuum/power consumption in battery-powered robots. In this paper, a CFD (computer fluid dynamics) modelling and optimization process of a robot adhesion system is carried out to determine the best performing configuration in terms of vacuum and power consumption. Analytical and numerical models were developed to predict the behaviour of the system for different configurations. The models were validated, using test rig measurements, by calibrating an arbitrary defined inlet height that simulates the leakage flow. Then, different geometric parameters were varied to determine the best performing configuration based on the vacuum/power consumption ratio value. The model presented in the paper was capable of predicting the behaviour of the system for different configurations, with a margin of error of 15% for the vacuum prediction and 25% for the motor power calculation. Finally, the model was used to optimize parameters of the system, like the number of blades of the impeller. The adhesion system was conceived for the modular autonomous climbing legged robot ROMERIN.

## 1. Introduction

Events such as the collapse of the Morandi bridge in Genoa in 2018 reflect the importance of regular maintenance and inspection of large infrastructure. Over the past half-century, the growth of civil infrastructure in many countries has been particularly intense. Consequently, those facilities require an ever-increasing expense for their inspection, replacement, or, if necessary, disassembly. The current conventional inspection methods are risky to the operators and the reliability and consistency of these processes reveal substantial variability in both the quality and quantity of the resulting assessments [1]. Robots can contribute to lower cost inspections, increasing the reliability of the obtained assessments and minimizing the risks to the operators [2]. Over the last few years, a major effort has been made to develop inspection robots, including ground-based robots, crawling and climbing robots, and unmanned aerial and marine vehicles [3].

Infrastructure inspections can be performed in various ways. Drone-based visual inspection is one of the most common, but it is not valid when there is no direct visual access to the area to be inspected or when it is not possible to fly a drone because of the narrowness or typology of the environment. In addition, the inspection usually requires much more than just seeing. There are many non-destructive techniques (e.g., ultrasonic, eddy-current, acoustic emission, etc.) that either require contact or proximity to the surface that is not possible to achieve while flying. Climbing robots, due to their ability to overcome these limitations, have increasingly become attractive for effective infrastructure inspection. In the past few years, a considerable number of climbing robots have been developed. A good summary of the technologies involved and the different approaches can be found in [4,5,6].

**Figure 1:**
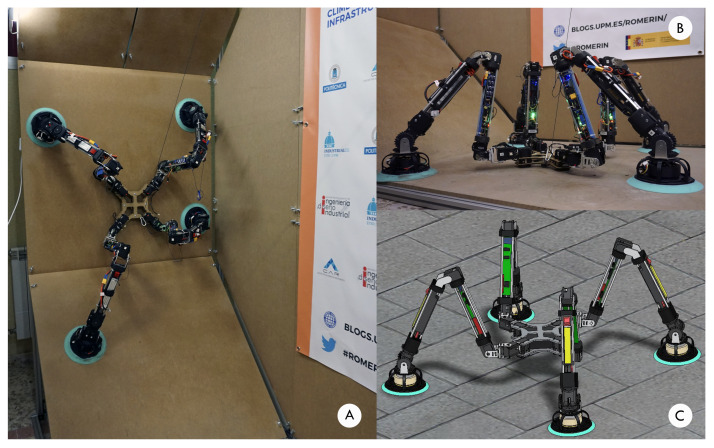
(**A**) Romerin modular robot stuck to the vertical wall of the test bench. (**B**) View of the robot starting up on the ground. (**C**) Romerin virtual model.

ROMERIN (Figure 1) is a new concept of a fully modular autonomous climbing legged robot designed to perform inspection tasks in civil infrastructure (i.e., cooling towers, tunnels, bridges, …) [7]. Its main task is to carry inspection equipment like 3D cameras or LIDAR sensors. It can be composed of different pair of legs configurations (4,6,8) equipped with suction systems that allow the robot to move over walls and ceilings. As it is modular, each leg works independently and consequently, it generates the necessary vacuum to be attached to the walls. Therefore, it does not have a pneumatic umbilical cord, as other robots have [8].

In this paper, we present the design procedure of the suction cups that has been applied to the modular climbing robot ROMERIN. The suction cup is placed at the end of the leg for sticking to walls or ceilings. To create the needed vacuum, every suction cup is equipped with its own centrifugal impeller and motor. This configuration has the main advantage of a better adhesion capacity in the presence of cracks or defects or on rough surfaces. The fact that the suction is generated independently in each leg avoids the effect of pressure loss in the entire robot when one of the suction cups is placed in an area with excessive air loss [9]). The design is optimized to achieve a power consumption as low as possible, while maintaining acceptable values of the vacuum. Therefore, the rotational speed can be limited to lessen the needed electrical power.

The main contribution of this article is to show the optimization process that leaded to the design and construction of the suction cup and the presentation of the obtained results.

The paper is structured as follows: in Section 2, an overview of the related work is given. In Section 3, the starting point and the followed methodology are shown. Section 4 is dedicated to describing the modelling and optimization process. In Section 5, the numerical model is validated. Finally, the optimization results are presented in Section 6.

## 2. Related Work

Climbing robots could be clearly differentiated based on two principles: the type of locomotion on which they are based and the adhesion system that they used. A good summary of the technologies involved in climbing robots and the different approaches they use can be found in [10]. Most climbing robots are based either on the use of suction or magnetic forces, or gripping mechanisms. The Magneto robot [11], besides being a very recent example of this last type of robots, is especially interesting because of the similarity with ROMERIN on the kinematics and some of the design concepts, like, for example, the 3 DOF compliant foot. The use of this type of joint at the end of the legs is important, since it avoids the transmission of high torques to the grip, as will be explained later. Nevertheless, the use of magnetic systems considerably narrows the application field and is not considered for infrastructure inspection.

In gripping mechanisms, the recent proliferation of electrostatic systems based on the imitation of gecko [12] or microspines, which employ arrays of miniature spines, that catch on surface asperities [13] or dry adhesives, is noteworthy [14]. However, these gripping mechanisms have the drawback of not being able to carry large weights. This makes them unsuitable for carrying inspection equipment.

Finally, regarding the use of suction forces, cleaning robots use a body adhesion system (usually suction by means of turbines or regenerative vacuum pumps) together with an independent locomotion system, such as wheels, that passively maintain contact with the surface. It has the drawback that it is not suitable for irregular surfaces.

Adhesion methods based on air pressure include vacuum adhesion, Bernoulli’s principle-based adhesion, vortex effect adhesion and aerodynamic pressure [15].

The vacuum adhesion mechanism is one of the most widely used methods in climbing robots. In this case, the adhesion force can be generated both actively and passively. The passive vacuum generation adhesion method does not require energy to generate the adhesion force. Vacuum cups are used and require a force to adhere or detach from the surface of interest. These forces are generated by the motion mechanism designed specifically for the robot. In this group, the robot developed by Yoshida et al. [16] stands out, where conventional vacuum cups are used, integrated into two guide belts driven by a motor. This method allows the use of the displacement mechanism to generate the vacuum needed to scale vertical walls, but, as a main disadvantage, the capacity of adhesion generation is very low and is reserved only for lightweight prototypes.

For active generating mechanisms, vacuum is achieved by using a vacuum generator, which may or may not be integrated into the robot. In this mechanism, the amount of vacuum that can be generated is much higher than for passive adhesion, enabling the possibility of using it in heavier models.

Due to the irregularities of the surfaces, losses can be generated in the seal of the suction cup, that can greatly reduce the amount of vacuum generated. For this reason, the robot presented in [17] consists of an arm that has several suction cups incorporated at each of its ends. The adhesion system used is made up of an arrangement of three suction cups in a triangular shape, a connector, a support plate, a vacuum pump, a pressure sensor, a two-way vacuum solenoid valve and a non-return valve. The main disadvantage of this mechanism is the amount of extra weight to be added to the robot.

Bernoulli’s principle is widely used in industrial robots for non-contact gripping operations, but the use of this principle to hold a robot on a vertical surface is relatively new. As an example of this method, the robot presented in [18] has the capacity to move in any direction and overcome small obstacles such as the joints between the tiles of a wall or the cracks of a tree. The robot is composed of two modules of suction (based on the principle of Bernoulli) at both ends of the robot and two wheels driven by DC motors. The robot has a payload of 0.5 kg with an own weight of 0.234 kg.

The vortex effect adhesion method consists of creating a rotating column of air by rotating a rotor that is installed in a closed cavity of a vacuum (suction) chamber. The rotation forms an area with reduced pressure that makes the robot stick to the surface. It is a very interesting method when using low consumption motors that allow to control the pressure according to the speed of rotation at low cost. Within this group, the City-Climber climbing robot [19] uses a rotor driven by an electric motor that, when rotating, generates a low-pressure area enclosed by a flexible vacuum chamber to adhere to a variety of surfaces. This method presents problems when working on surfaces with considerable levels of dirt, as they could become encrusted within the rotor, causing a decrease in the performance of the system. In addition, it should be noted that when the rotor rotates at high speeds, large amounts of noise can be generated.

Finally, the aerodynamic pressure adhesion mechanism generates the pressure by rotating a propeller. The main example of this type is the climbing robot for anti-terrorist and rescue exploration tasks developed by Liu et al. [20], which is composed of a suction cup, a pair of wheels, a flexible sealing ring and a propeller. The operating principle of the robot is based on the critical suction method, which consists of the generation of two types of adhesion forces caused by the generation of vacuum in the suction cup and the thrust force of the propeller, respectively. This causes the robot to be pushed towards the wall without the need to generate physical contact between the suction cup and the wall, thus providing greater versatility in adaptation. The main disadvantages of this method are high energy consumption and noise.

The ROMERIN robot adhesion system design must be adapted to a climbing robot that uses legs as locomotion mode. For this reason, it was decided to develop a hybrid vacuum generation system, using the active vacuum generation adhesion mechanism and the vortex effect. For this purpose, a design was developed, consisting of a centrifugal-type rotor, driven by a brushless motor, which is adapted to a vacuum-generating suction cup. The operating principle is then based on the generation of vacuum in the internal cavity of the suction cup, through the generation of a vortex caused by the rotation of the rotor. By using this method, it is sought to obtain good adhesion forces while maintaining a low energy consumption.

## 3. Starting Point and Methodology

The objective of the article is to present a modelling and optimization procedure for an adhesive system of a modular climbing robot. Two prototypes will be used for this purpose: a test bench prototype (TBP) and an onboard robot prototype (ORP).

The TBP is a general-purpose prototype (not for the robot) based on traditional air suction systems designed to test concepts and physical principles. It is the starting point of the experiments.

The ORP is a scaled down version of the TBP designed for the modular robot. It will be the first version of the adhesive system on the robot and it is based on more evolved suction cups.

**Figure 2:**
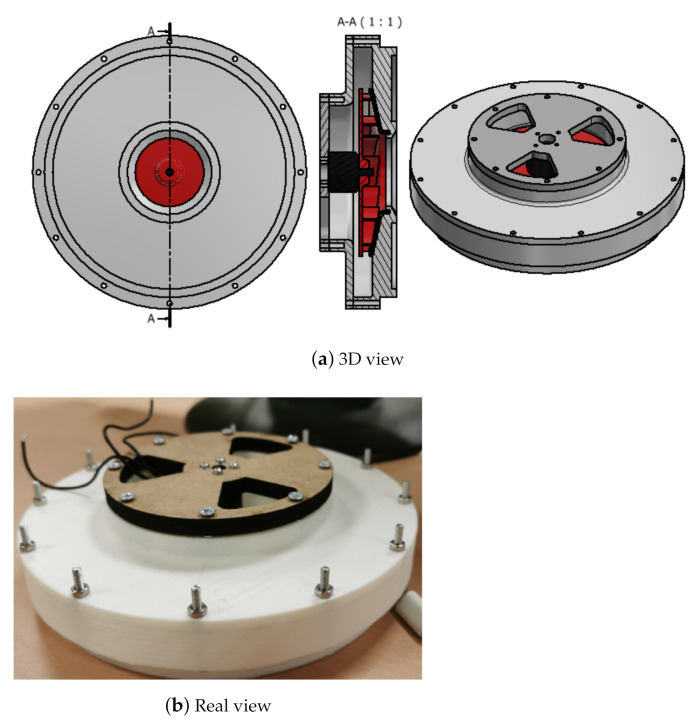
Suction system: housing, impeller (in red) and motor cover.

The design of the suction system for both the TBP and the ORP consists of a housing (with integrated diffuser), impeller (with integrated motor), motor cover, coupling to the robot’s leg and suction cup, as shown in Figure 2.

The methodology is composed of the following actions: in the first place, a model that describes the desired behaviour of the suction cups is built (Section 4). Then, this model is validated using both prototypes, the TBP and ORP (Section 5). This is an iterative process, meaning that the results obtained in the TBP are used to improve the ORP. Finally, the prototypes are optimized to reduce the power consumption while maintaining the adhesion performance (Section 6).

The main elements are described below: the TBP, the ORP and the test rig used to test the two prototypes.

### 3.1. TBP Design

As a starting point, it was decided to design a model of centrifugal vacuum pump inspired by traditional air suction systems; consisting of a centrifugal impeller driven by a brushless motor that is integrated into housing. To carry out the design of the main parts, diverse results extracted from [21,22,23] are integrated, since the existing relationships between the different geometric parameters of the rotor and the physical variables of interest are evidenced.

**Figure 3:**
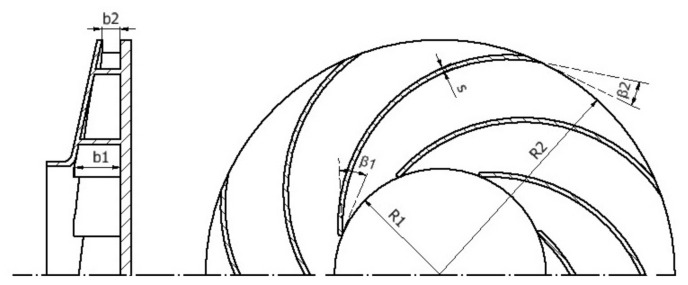
Impeller main geometric parameters.

For the impeller design (Figure 3), several design recommendations regarding the number of blades (*z*), blade angles ($\beta_{1},\beta_{2}$), widths ($b_{1},b_{2}$), diameters ($d_{1},d_{2}$), blade shape and casing type were used to develop the first model. Finally, bearing in mind the limitations set by the manufacturing process used, the geometric values were set, as shown in Table 1. The motor that has been used is the DYS MR2205.

Finally, the design of the casing was conducted by trying to reduce the air leakage through the gap that exist between the fan and the shell. To do so, the gap was set at a small yet safe value to avoid touching. In addition, at the eye of the casing, an inward lip towards the eye of the impeller is used to direct the flow to the impeller.

### 3.2. ORP Design

This prototype is a reduced version of the TBP designed to be mounted on the modular robot. The development of the ORP is carried out in parallel to that of the TBP, using the experimental results obtained with the TBP tests. Therefore, the design of the ORP is similar to that of the TBP shown in the previous section, but reducing its dimensions by applying the laws of similarity of turbomachines. The physical dimensions of this prototype can be seen in Table 1. The motor used is DYS BE1806-2300KV.

### 3.3. Test Rig

**Figure 4:**
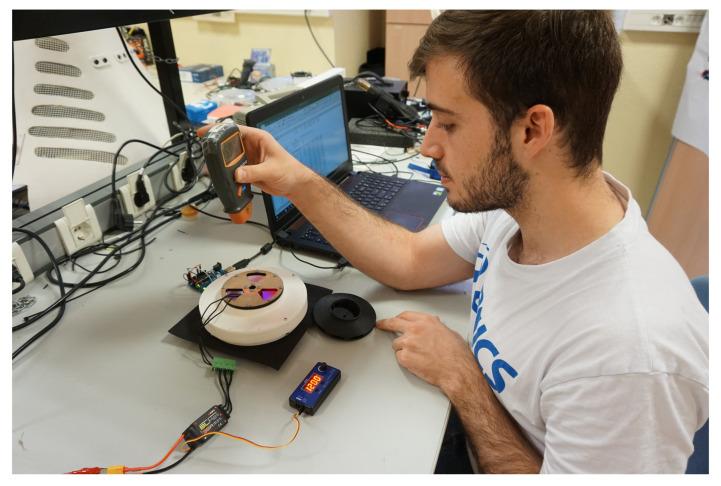
Test rig used to perform experimental measurements.

To measure the vacuum pressure generated inside the suction cup, as well as the impeller rotational speed and motor electrical power, a test rig (Figure 4) was built using the following components:

- A 3D printed base, integrated with a BMP 180 barometric pressure sensor, and connected to a microcontroller, to read the absolute pressure value reached inside the suction cup chamber.
- A digital laser tachometer to measure the impeller rotational speed.
- A variable voltage source to adapt the input voltage and show the consumed current reading to measure the motor power.
- A potentiometer, connected to the motor ESC, to vary the rotational speed of the impeller.

Once the measurements were completed, the values of vacuum (Δp), motor power consumption ($\dot{W}$), resulting adhesive force ($F_{p}$) and vacuum-consumption ratio (η) were calculated using Equations (1)–(4), respectively.

(1) $$\Delta p=p_{measured}-p_{atm}$$

(2) $$\dot{W}=V\left(I-I_{0}\right)$$

(3) $$F_{p}=\Delta p\pi {R_{cup}}^{2}$$

(4) $$\eta =\frac{\Delta p}{\dot{W}}$$

where ($p_{measured}$) is the pressure value measured inside the suction cup cavity, ($p_{atm}$) is the atmospheric pressure, ($R_{cup}$) is the suction cup radius, (*V*) is the voltage consumed by the motor, (*I*) represents the current consumed by the motor and ($I_{0}$) the no-load current, respectively.

## 4. Model Definition

Given that the robot is powered exclusively by Li-Po batteries, the suction system’s energy consumption becomes a major concern, considering that most of the adhesion systems developed for climbing robots based on vacuum generation are continuously connected to sources of energy. Moreover, the suction system should be capable to generate enough vacuum to allow the robot to carry a definite amount of payload and ensure proper functioning in irregular vertical surfaces, where the air leakage flow will directly affect vacuum production. For these reasons, an optimization process is carried out, focused on:

- Allowing smooth operation of the robot in rough surfaces, such as concrete, considering the leakage airflow caused by irregular contact between the suction cup sealing ring and the surface wall irregular shape.
- Producing enough suction power to carry definite amounts of payload in addition of the robot arm overall weight.
- Reducing the system power consumption to ensure longer robot operation time.

The process of modelling the system in order to carry out the optimization process is described below.

### 4.1. Analytical Model

As a first step, an analytical model of the system is developed to guide the optimization process to achieve successful results. In the following, the formulation used in the development of the physical and fluid model of the system, as well as different concepts extracted from turbomachinery theory, are presented. It should be noted that the application of the presented turbomachines concepts is to estimate the motor power and to adjust the geometric parameters of the system as the experimental results were obtained.

#### 4.1.1. Physical Model

**Figure 5:**
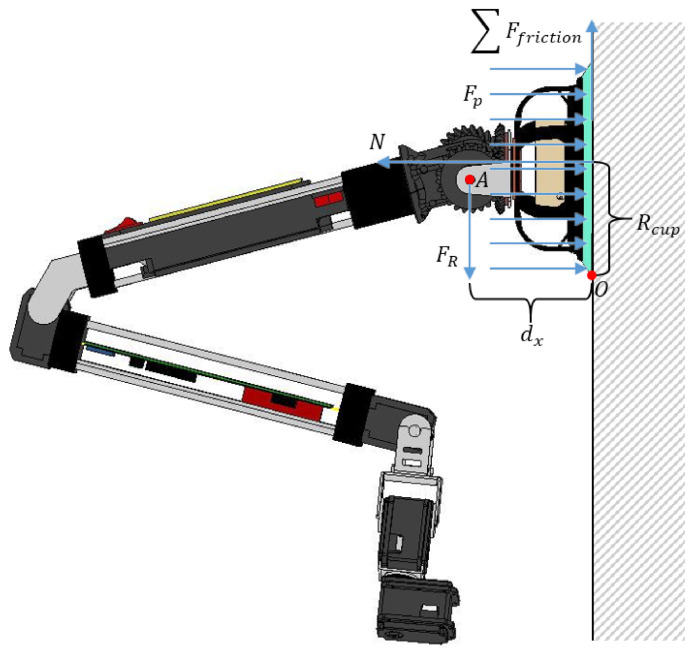
Physical model of ROMERIN robot with vacuum generation system.

For the robot to be able to climb a vertical wall (Figure 5), the suction system must generate sufficient adhesive force to support the full weight of the robot, preventing it from slipping and overturning. Analysing both critical conditions, we can obtain relationships for the robot total weight and the vacuum pressure requirements.

For the slipping condition, the robot should not fall off due to its overall weight. Therefore, the suction system should generate sufficient adhesive force to keep the robot attached to the wall. Looking then at the force diagram, the following relationships can be deduced:

(5) $$N=F_{p}=\Delta pA_{cup}=\pi {R_{cup}}^{2}\Delta p$$

(6) $$\Sigma F_{friction}=F_{R}$$

where:

- *N*: Counterforce of the wall surface, measured in Newtons.
- $F_{R}$: The force of the weight of the robot incorporating the payload to be transported in it measured in kilograms, and:
  (7) $$F_{R}=g\left(m_{Robot}+m_{Payload}\right)$$
- $F_{p}$: The adhesive force generated by the negative pressure inside the suction cup [N].
- $R_{cup}$: The radius of the suction cup [m].
- $\Sigma F_{friction}$: The summation of friction forces between the robot suction cup and the wall surface, and:
  (8) $$\Sigma F_{friction}\leq \mu_{e}N$$
- $\mu_{e}$: Static friction coefficient of the wall surface.

On the other hand, to avoid overturning, the moment created by the suction force should be greater than the resultant force moment, due to the robot overall weight. As shown in Figure 5, the resultant force $F_{R}$ is applied at point A. This is due to the fact that at this point there is an spherical joint with reversible servomotors, which does not allow the transmission of momentum to the suction cup, but rather the transmission of forces. Therefore:

(9) $$F_{p}R_{cup}=d_{x}F_{R}$$

where $d_{x}$ is the horizontal distance measured from the wall surface to the robot wrist.

Substituting then (7) & (8) into (5), (6) & (9), and solving for the pressure gradient leads to:

(10) $$\Delta p\geq \frac{g(m_{Robot}+m_{Payload})}{\mu_{e}\pi {R_{cup}}^{2}}$$

(11) $$\Delta p\geq \frac{gd_{x}\left(m_{Robot}+m_{Payload}\right)}{\mu_{e}\pi {R_{cup}}^{3}}$$

To avoid slipping (10) and overturning (11), the vacuum system must provide sufficient suction power to satisfy both conditions (12). Table 2 shows the estimated minimum values of vacuum pressure to be achieved, considering that the robot is holding still in a concrete wall ($\mu_{e}$ ≈ 0.6).

(12) $$\Delta p\geq max\left(\frac{g(m_{Robot}+m_{Payload})}{\mu_{e}\pi {R_{cup}}^{2}},\frac{gd_{x}\left(m_{Robot}+m_{Payload}\right)}{\mu_{e}\pi {R_{cup}}^{3}}\right)$$

#### 4.1.2. Fluid Model

For the fluid model, an approximate method is used to estimate leakage flow due to the irregular contact between the wall surface and the suction cup sealed ring.

**Figure 6:**
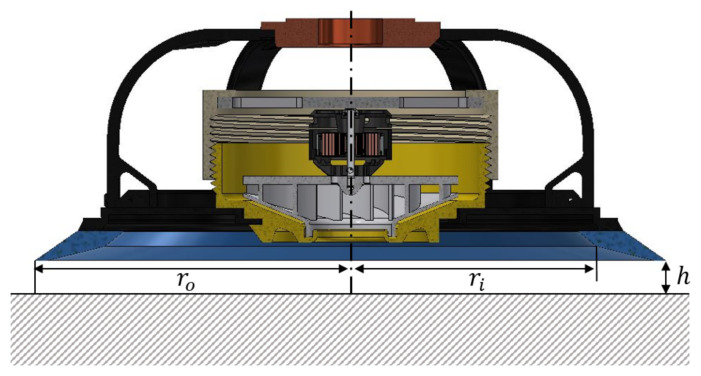
Fluid model of the robot suction system.

Considering the fluid model proposed by [24], it can be assumed that it exist a gap between the suction cup sealing ring and the surface wall (Figure 6) to obtain a relationship between the vacuum pressure, air leakage and the sealing ring radius, as shown in (13).

(13) $$Q=\frac{\pi h^{3}\Delta p}{6\mu ln\left(\frac{r_{o}}{r_{i}}\right)}$$

where:

- *Q*: Airflow leakage [*l*/s].
- *h*: The gap between the suction cup sealing ring and the surface wall [mm].
- $r_{o},r_{i}$ : The suction cup sealing ring inner and outer radius, respectively [mm].
- μ: Air dynamic viscosity [Pa-s]

#### 4.1.3. Turbomachine Motor Power Calculation

The motor power is the power consumed by the motor to turn the impeller. It can be defined as the sum of the shaft power and power loss due to inefficiencies in converting electric energy into kinetic energy [25]. The motor power ($\dot{W}$) can be calculated dividing the shaft power by the motor efficiency, as shown in (14).

(14) $$\dot{W}=\frac{P_{shaft}}{\eta_{m}}=\frac{M_{impeller}\omega }{\eta_{m}}=\frac{\frac{2\pi }{60}nM_{impeller}}{\eta_{m}}$$

where ($M_{impeller}$) is the impeller’s torque, (*n*) is the rotational velocity of the impeller, measured in RPM, and ($\eta_{m}$) represents the motor efficiency.

#### 4.1.4. Turbomachines Similarity Laws

The use of dimensionless performance variables for pumps is desirable in a systems analysis, since it allows for greater flexibility in the use of its performance data. In this case, it can be shown in (15) and (16) how the turbomachine performance for incompressible fluids can be characterized in terms of power and pressure.

(15) $$C_{\dot{W}}=\frac{\dot{W}}{\rho \omega^{3}d^{5}}$$

(16) $$C_{\dot{p}}=\frac{\Delta p}{\rho \omega^{2}d^{2}}$$

where (ω) is the impeller rotational speed, (*d*) is the diameter and ($C_{\dot{W}}$) and ($C_{\dot{p}}$) represents the power and pressure coefficients, respectively.

The turbomachines laws of similarity are used to predict the behaviour of a machine of different size, but geometrically similar to another whose behaviour (pressure, power, etc.) is known. For the following work, the laws of power and pressure variation similarity were applied, where the power and pressure coefficients of two geometrically similar pumps are equalized, yielding the following relationships:

(17) $$\frac{{\dot{W}}_{m}}{{\dot{W}}_{p}}={\left(\frac{d_{m}}{d_{p}}\right)}^{5}{\left(\frac{n_{m}}{n_{p}}\right)}^{3}$$

(18) $$\frac{\Delta p_{m}}{\Delta p_{p}}={\left(\frac{d_{m}}{d_{p}}\right)}^{2}{\left(\frac{n_{m}}{n_{p}}\right)}^{2}$$

where the subscripts *m* and *p* represent the model and the prototype, respectively.

### 4.2. Numerical Model

To carry out the numerical simulations, a commercial finite volume-based Navier—Stokes solver was used, ANSYS Fluent 18. The numerical model used is described below, divided into the phases of fluid domains definition, grid generation, boundary conditions definition and turbulence model selection.

#### 4.2.1. Fluid Domains Definition

**Figure 7:**
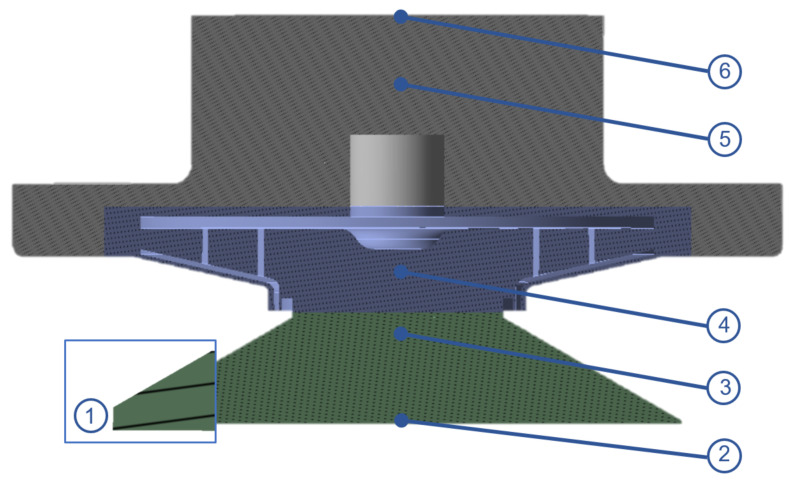
Suction cup Flow domains: (**1**) Inlet, (**2**) Surface wall, (**3**) Suction cup cavity, (**4**) Impeller rotational domain, (**5**) Casing and (**6**) Outlet.

To develop a correct numerical simulation (computer fluid dynamics (CFD)) of the system, it is necessary first to define the flow domain. Using Inventor CAD software, the flow domain was modelled as the integration of the impeller with all blade passages, the suction cup including an inlet face to mimic the gap between the sealing ring and the surface wall, the casing volume and the outlet face (Figure 7).

The reason for including the suction cup cavity and the casing domain in the simulation is that in this way one can directly compare the results obtained from the numerical simulation with the measurements from the test rig. By doing so, the assumed inlet height can be calibrated to match the experimental results to validate the model.

#### 4.2.2. Grid Generation

**Figure 8:**
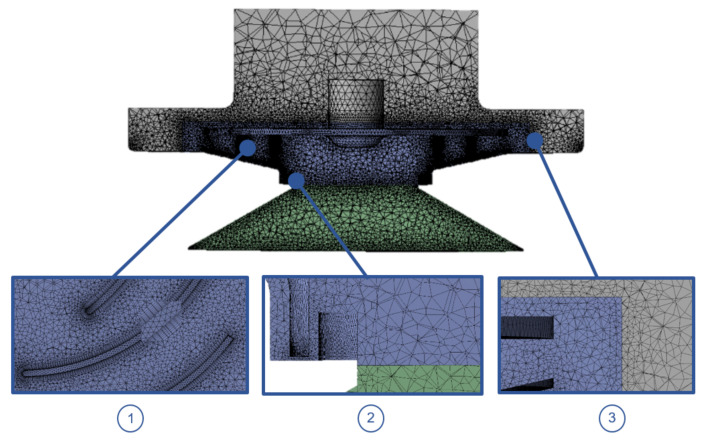
Grid details: (**1**) Tetrahedral and prism elements in the impeller blades. (**2**) Mesh interface between the suction cup and impeller domains. (**3**) Mesh interface between the casing and impeller domains.

The grids were generated using ANSYS 18.0 Fluent Mesh tool. They consist of tetrahedral and prism elements at the walls (Figure 8). The mesh for the TBP was modelled with 6,746,453 elements, and for the ORP, since it is smaller and therefore has a smaller volume, with 5,502,985 elements. Table 3 shows the quantity of elements used for each flow volume.

#### 4.2.3. Boundary Conditions Definition

For the boundary conditions, both inlet and outlet domains, shown in Figure 7, were set as pressure-based boundaries, with a value equal to the ambient pressure. Furthermore, the specified rotational speeds for the impeller in the fluid domain corresponded to different values extracted from the test rig measurements.

Since both suction cup and casing domains are stationary, a multiple-frame-of reference (MRF) numerical calculation was performed to simulate the rotation of the impeller. The interfaces between the different frames of reference were set as moving walls, with a relative rotational speed equal to 0. Moving walls boundary conditions refer to the class of grid connections where the mesh on either side of the two connected surfaces does not match (Figure 8). The flow properties at the interface between the stationary and rotary zones are translated directly into the MRF. In the approach of the method, there is no relative movement between the moving zone with respect to the adjacent zone, because the meshing is fixed for the calculation. This is analogous to freezing the movement of the moving part in a position and observe the instantaneous flow field with the rotor in that position. For this reason, the method is often referred to as “frozen rotor” [26].

According to Epple [22], the frozen rotor model has the advantages of being robust, using less computational resources than other frame change models, and being well suited for high blade counts. The main disadvantage of using this model is that it cannot model the transient effects at the frame interfaces. However, In this case, this is not necessary, because steady flow simulations were performed using a fixed speed of rotation.

#### 4.2.4. Turbulence Model Selection

The turbulence model applied was the shear stress transport (SST) k−ω model of Menter [27]. The model effectively blends the robust and accurate formulation of the (k−ω) model in the near-wall region with the free-stream independence of the (k−ϵ) model in the far field. The SST model performance has been studied in a large number of cases, and according to NASA’s memorandum [28], it can be considered as one of the most accurate models for aerodynamic applications.

Finally, to assure that the solution has converged, in addition to the mass, momentum, energy, turbulence kinetic energy, and turbulence frequency residuals, the torque of the impeller and the average absolute pressure in the wall surface were monitored.

## 5. Numerical Model Validation

Once the numerical model configuration is completed, it is important to assure that the results obtained from the simulations are capable to successfully predict the behaviour of the system.

Since the numerical model is an approximation of the test rig, it is necessary to adjust the arbitrary defined parameters using experimental measurements. For this reason, the results obtained on the test bench are presented below, followed by the calibration process and validity check of the numerical model.

### 5.1. Test Rig Measurements

The following are the results obtained when analysing the behaviour of the TBP and ORP models on the test bench, described in Section 3.2.

#### 5.1.1. TBP Design

Regarding the TBP results, shown in Table 4, it can be seen that the system is capable of generating large amounts of vacuum for relatively low rotational speeds. However, the electrical power consumption to achieve these values is excessive, causing the efficiency of the system to be low. For this reason, it was decided to carry out an optimization process, described in Section 6, focused on improving the vacuum/power consumption ratio.

#### 5.1.2. ORP Design

Subsequently for the ORP, it is important to emphasize that this design is a result of scaling the result obtained once the TBP is optimized. Hence, direct comparisons between TBP and ORP models should not be made, as they have considerably different sizes.

Analyzing, then ,the performance of the ORP (Table 5), it can be seen that the quantities of vacuum generated are maintained in an acceptable range of operation for the robot, while obtaining a much lower energy consumption for relatively higher rotational speeds. This results in a significant enhancement of the system’s efficiency. Nevertheless, this change is due in part to the considerable increase in the rotation speed of the rotor, which can augment the wear and tear of system components.

### 5.2. Model Validation

The numerical model validation process is carried out by calibrating the arbitrarily defined inlet height, as shown in Figure 6). This height is iterated until the average absolute pressure value, measured on the concrete wall surface where the suction cup is placed, matches the experimentally measured vacuum value. Additionally, it is established as a second condition that the calculated power, using the Equation (14), has a value close to the electrical power consumed during the experiment. It was considered a margin of error of 15% for the pressure estimation and 25% for the motor power calculation.

#### 5.2.1. TBP Model

Observing the results obtained for the TBP model (Table 6), it can be seen how an inlet height of 0.5 mm satisfies both conditions for the experimental point where the impeller is spinning at 18,500 RPM. However, to ensure that this inlet height is suitable for other cases, three more experimental points where simulated and compared.

**Figure 9:**
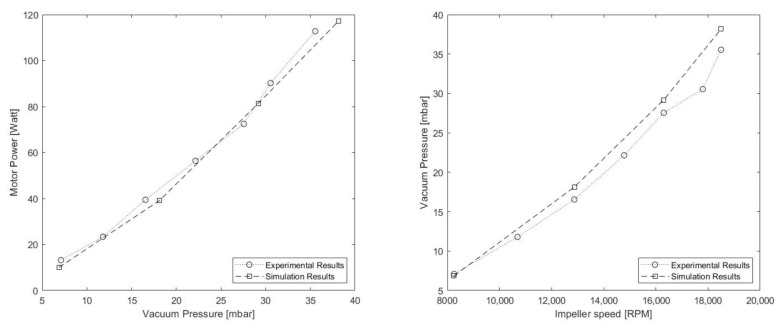
TBP simulation vs. experimental results graph: (**left**) vacuum pressure vs. motor power. (**right**) impeller speed vs. vacuum pressure.

After reviewing the results of the comparison (Table 7 and Figure 9), the numerical model proved that it is capable to predict the behaviour of the system with an acceptable margin of error. Nevertheless, it is worth noting that the inaccuracy of the model in higher RPM counts is usually caused by the increasing complexity and instability of the simulation to predict the behaviour of the flow in the interfaces between the rotational and static domains.

#### 5.2.2. ORP Model

In the same manner, the procedure was repeated for the ORP model. It is necessary to repeat the validation of a new model because the design suffers considerable changes in the size and shape of its components.

**Figure 10:**
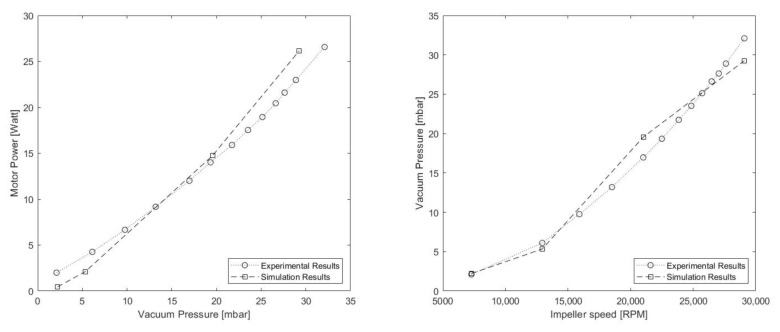
ORP simulation vs. experimental results graph: (**left**) vacuum pressure vs. motor power. (**right**) impeller speed vs. vacuum pressure.

The ORP inlet height was calibrated then analysing the case where the impeller spins at 29,090 RPM (Table 8) with a resulting value of 0.13 mm. Considering then 3 more experimental points, as shown in Table 9 and Figure 10, the new model was validated. The results show how the error stays within the limits considered by the study.

## 6. Optimization Results and Discussion

Once the experimental results of the TBP were analyzed (Table 4), it was evidenced that the system was capable of reaching acceptable vacuum values. However, the consumption of energy was very high, diminishing, in great measure, the time of operation of the robot. For this reason, it was decided to carry out an optimization process focused on reducing energy consumption, while maintaining acceptable vacuum values. To do so, a sensitivity study of different geometrical parameters of the rotor was conducted, using the validated numerical model, to determine a more efficient configuration. Finally, the system main components were scaled using turbomachines similarity rules, since the previous size was larger than needed.

### 6.1. Impeller Geometrical Parameters Sensitivity Study

**Figure 11:**
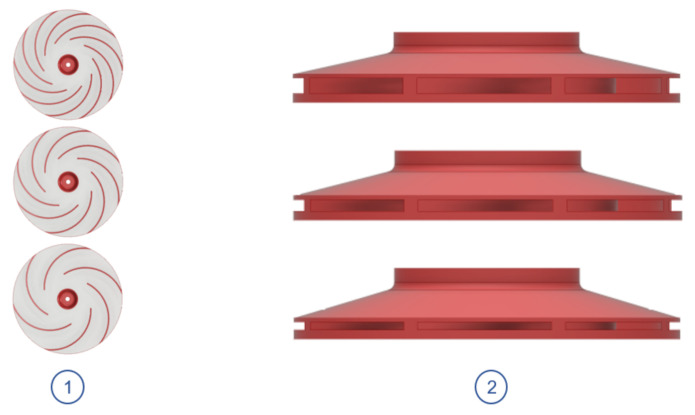
Impeller geometric parameters sensitivity analysis: (**1**) number of blades. (**2**) Outlet width.

Based on previous work experience, it was decided to vary the number of blades and outlet height, as shown in Figure 11, while maintaining the rest of geometrical parameters fixed. Thus, independent analyses were performed for each parameter to measure the impact in vacuum and motor power consumption. The behaviour of the system was then evaluated for three different experimental points in terms of the amount of vacuum generated and the calculated motor power consumption.

#### 6.1.1. Number of Blades

For the number of blades, it was decided to analyze an impeller with 6, 8 and 10 blades, respectively. The TBP tested impeller is the one with 10 blades. Changing the number of blades can be interpreted as changing the aspect ratio of the blade channels on the impeller, either by increasing (less blades) or decreasing (more blades) the flow space between blades.

Observing the results obtained for the number of blades (Table 10), it was determined that the increase in the number of blades causes an increment in the vacuum pressure generation. This is do to the fact that as the impeller blade channels are smaller, the flow guidance is improved and the slipping effects are reduced. In contrast, the motor power consumption increases, since this reduction introduces more losses in the internal flow due to friction and mixing flows. This effect can be evidenced for higher RPM values, where the introduced losses for higher blade counts increases the motor power consumption, without a considerable increase in vacuum pressure. Finally, the impeller with 6 blades is selected, since it achieves the best vacuum/consumption ratio for all the cases.

#### 6.1.2. Impeller Outlet Width

On the other hand, for the impeller outlet width, values of 2, 3 and 4 mm were considered. The TBP tested impeller is the one with an outlet width of 4 mm.

Looking at the results obtained for the impeller outlet width (Table 11), it can be seen how both vacuum pressure and motor power decreases as the outlet width is reduced. In contrast, the vacuum/power ratio increases as the output width is smaller. Comparing the different outlet widths, it was determined that a value between 2 and 3 mm will improve the performance of the model, since it presents a higher ratio value and maintains great vacuum values. From this range, a value of 2.5 mm was finally selected after considering the additional complexity that a lower value could introduce to the manufacturing process.

Once the sensitivity analysis was completed, the system components were scaled up by applying the laws of similarity for turbomachines. Considering that:

- The experimental point for the TBP is the one where the impeller spins at 18,500 RPM.
- The new model will achieve the same amount of vacuum, with the impeller spinning at 30,000 RPM with a maximum motor power consumption set at 35 Watt.

Then, the new diameter of the impeller was calculated using Equations (17) and (18). Finally, applying the modifications obtained from the optimization process and maintaining the relationships between the others geometric variables of the rotor, the dimension of the new impeller were set, as shown in Table 12.

### 6.2. CFD Results

Having tested and validated both prototypes models (Section 5.1 and Section 5.2), a qualitative study of the behaviour of the systems was carried out using the CFD simulations results, commenting on the different physical phenomena that are illustrated in the absolute pressure, velocity and streamlines contour plots. The contour plots correspond to those in which the impeller was rotating at 18,500 RPM for the TBP model, and at 29,090 RPM for the ORP model.

#### 6.2.1. TBP Model

**Figure 12:**
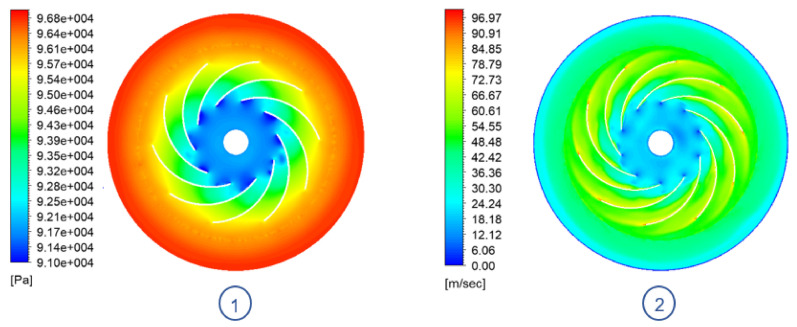
TBP model impeller plane view @ 18,500 RPM: (**1**) Absolute pressure contour. (**2**) Velocity contour.

Studying the behaviour of the flow that moves through the impeller, it is possible to observe the flow separation that occurs at the leading edge of each blade (Figure 12), which generates low pressure bubbles in the flow channels of each blade. Next, as the flow moves through the channels, the pressure gradient between the inlet and outlet of the rotor is evidenced. Finally, the flow continues to increase its pressure once it leaves the impeller because it abruptly reduces its velocity by colliding with the casing.

**Figure 13:**
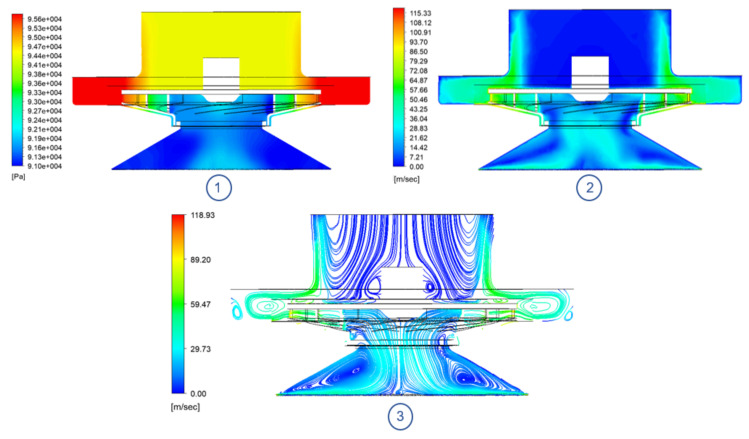
TBP model median plane @ 18,500 RPM: (**1**) Absolute pressure contour. (**2**) Velocity contour. (**3**) Streamlines.

Analyzing, then, the behaviour of the system from a mid plane view (Figure 13), the air suction phenomenon, caused by the rotation of the impeller, can be noticed. As the impeller turns at high speed, it creates a vortex inside the suction cup cavity generating two large low pressure bubbles on the side walls. At the same time, the leakage flow enters the system through the calibrated inlet height. Lastly, turbulent eddies are generated in the region between the impeller outlet and the casing, hindering the evacuation of the flow.

#### 6.2.2. ORP Model

After applying the changes derived from the sensitivity analysis and scaling the prototype, it can be seen how the ORP is capable of generating similar amounts of vacuum as the TBP model. Since the impeller is rotating at a higher speed rate, it is capable of compensating the amount of work done on the flow using a smaller diameter. However, this causes the system losses to be greater, as the flow is accelerated to a greater extent.

**Figure 14:**
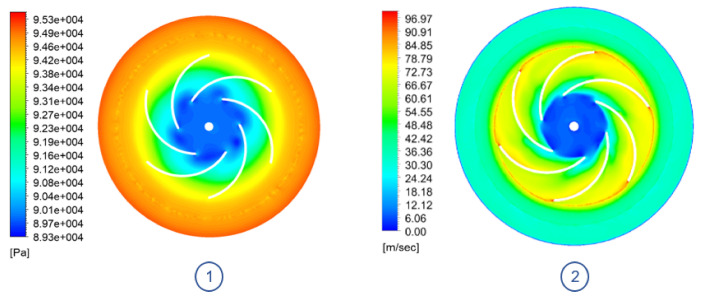
ORP model impeller plane view @ 29,090 RPM: (**1**) Absolute pressure contour. (**2**) Velocity contour.

In this configuration, shown in Figure 14, it can be seen how the appearance of low pressure bubbles on the leading edge of the blades is corrected, thus improving the air intake. However, as the flow circulates through the channels, its speed increases rapidly, reaching high tip speeds values. This can be an issue because the higher the speed at the tip of the impeller, the more energy that is imparted to any particle that is suspended within the fluid. This energy can then cause damage to anything it impacts (i.e., impeller blades, casing).

**Figure 15:**
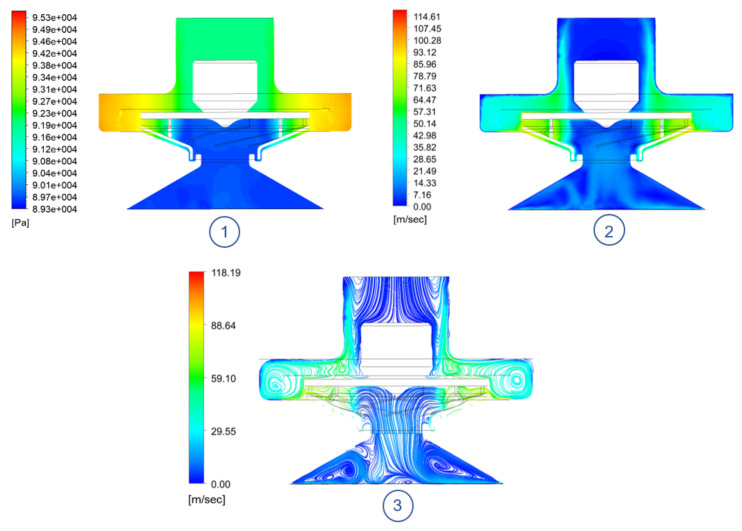
ORP model median plane @ 29,090 RPM: (**1**) Absolute pressure contour. (**2**) Velocity contour. (**3**) Streamlines.

On the other hand, in the mid-plane view (Figure 15), one can observe how the low pressure bubbles in the suction cup are larger, since the calibration height is lower than in the TBP model, causing less air to enter the system. In addition, a turbulent vortex continues to be observed between the rotor outlet and the casing, as in the initial model, generated by the shock of the high-speed flow impacting the housing. However, it was determined that this effect did not significantly affect the overall performance of the system.

## 7. Conclusions

In this work, a CFD modelling and optimization process of adhesion systems based on active vacuum generation using air suction was presented. It was shown how to validate the numerical model using test rig measurements and to adjust different geometric parameters of the impeller, via simulation, to improve the vacuum/power consumption ratio. As an example, the effect of varying the number of blades and the impeller outlet width was studied.

For the number of blades, three different configurations were evaluated (i.e., 6, 8, 10) in terms of vacuum generated and power consumption. It was determined that a higher blade count improves the flow guidance, but adds more friction losses, which increases the motor power consumption. It was concluded that a blade count of six was the one with the best performance, since it presents the best vacuum/consumption ratio for all the studied cases.

Then, for the impeller outlet width, the same procedure was repeated, using values of 2, 3 and 4 mm, respectively. It was determined that the vacuum/power ratio is improved as the outlet width is reduced. However, this improvement is diminished when the output width is too small. Considering the need to lower the power consumption to acceptable values and the additional complexity that a smaller value could add to the manufacturing process, the selected output width resulted in a value of 2.5 mm.

Once the sensitivity analysis was completed, the impeller design was modified by applying the changes derived from the study. In addition, the system was scaled up, applying turbomachine similarity rules, to reduce the motor power consumption while maintaining acceptable vacuum values.

Finally, the new design was tested and modelled to observe the changes in the behaviour of the flow. Here, it was evidenced how at the inlet of the impeller the generation of low pressure bubbles in the leading edge of the blades was corrected. Nevertheless, it an increment in the outlet speed of the impeller was observed, due to the higher rotational speed, that can increase the risk of damaging the parts of the system with any particle that could be suspended within the fluid.

## Figures and Tables

**Table 1:** Main dimensions of the test bench prototype (TBP) and an onboard robot prototype (ORP) impellers design.

Parameter	TBP Value	ORP Value	Units
$\beta_{1}$	25	25	deg
$\beta_{2}$	15	15	deg
$d_{1}$	46	23	mm
$d_{2}$	100	60	mm
$b_{1}$	10	10	mm
$b_{2}$	4	3	mm
*z*	10	10	-
*s*	1	1	mm

**Table 2:** Vacuum pressure requirement for different payload values.

Payload [gr]	Δp Slipping [mbar]	Δp Overturning [mbar]
0	14.30	12.87
125	15.32	13.79
250	16.34	14.70
375	17.35	15.62
500	18.37	16.53
625	19.39	17.45
750	20.40	18.36
875	21.42	19.28
1000	22.44	20.19

**Table 3:** Mesh elements in detail for each flow domain.

Fluid Domain	TBP Model	ORP Model
Suction Cup	2,180,838	1,104,896
Impeller	3,875,564	3,832,417
Stator	690,051	565,672
Total	6,746,453	5,502,985

**Table 4:** Test rig measurements for the TBP.

RPM	V	*I* [Amp]	$\dot{W}$ [Watt]	Δp [mbar]	$F_{p}$ [N]	η [mbar/Watt]
8256	16.11	0.82	13.21	7.10	14.20	0.54
10,692	16.11	1.45	23.36	11.80	23.60	0.51
12,870	16.11	2.45	39.47	16.55	33.10	0.42
14,782	16.11	3.5	56.39	22.15	44.30	0.39
16,300	16.11	4.5	72.50	27.55	55.10	0.38
17,800	16.11	5.6	90.27	30.55	61.10	0.34
18,500	16.11	7	112.77	35.55	71.10	0.32

**Table 5:** Test rig measurements for the ORP.

RPM	V	*I* [Amp]	$\dot{W}$ [Watt]	Δp [mbar]	$F_{p}$ [N]	η [mbar/Watt]
7281	14.3	0.24	2.00	2.11	4.22	1.05
12,940	14.2	0.4	4.26	6.11	12.22	1.43
15,907	14.2	0.57	6.67	9.76	19.52	1.46
18,516	14.1	0.75	9.17	13.20	26.4	1.44
21,017	14.3	0.94	12.01	16.97	33.94	1.41
22,499	14.3	1.08	14.01	19.34	38.68	1.38
23,849	14.2	1.22	15.90	21.74	43.48	1.37
24,854	14.25	1.33	17.53	23.53	47.06	1.34
25,728	14.25	1.43	18.95	25.14	50.28	1.33
26,480	14.3	1.53	20.45	26.63	53.26	1.30
27,046	14.4	1.6	21.60	27.63	55.26	1.28
27,622	14.45	1.69	22.98	28.89	57.78	1.26
29,090	14.6	1.92	26.57	32.10	64.20	1.21

**Table 6:** Inlet height calibration for the TBP numerical model @ 18,500 RPM.

*h* [mm]	Δp [mbar]	Δp Error%	$M_{impeller}$ [N.m]	$\dot{W_{c}}$ [Watt]	$\dot{W_{c}}$ Error%
0.14	46.79	31.63%	0.0512	104.18	7.62%
0.20	44.88	26.23%	0.0596	121.29	7.56%
0.50	38.17	7.37%	0.0576	117.26	3.98%

**Table 7:** TBP numerical model comparison with different experimental points.

RPM	Δp [mbar]	Δp Error%	$M_{impeller}$ [N.m]	$\dot{W_{c}}$ [Watt]	$\dot{W_{c}}$ Error%
8256	6.87	3.22%	0.0111	10.06	23.85%
12,870	18.12	9.46%	0.0277	39.15	0.81%
16,300	29.18	5.90%	0.0454	81.42	12.31%

**Table 8:** Inlet height calibration for the ORP numerical model @ 29,090 RPM.

*h* [mm]	Δp [mbar]	Δp Error%	$M_{impeller}$ [N.m]	$\dot{W_{c}}$ [Watt]	$\dot{W_{c}}$ Error%
0.14	24.19	24.64%	0.0068	21.79	18.00%
0.13	29.25	8.8%	0.0078	23.76	6.11%

**Table 9:** ORP numerical model comparison with different experimental points.

RPM	Δp [mbar]	Δp Error%	$M_{impeller}$ [N.m]	$\dot{W_{c}}$ [Watt]	$\dot{W_{c}}$ Error%
7281	2.22	5.06%	0.0020	1.588	20.68%
12,940	5.34	12.64%	0.0023	3.130	22.84%
21,017	19.56	15.26%	0.0061	13.40	22.68%

**Table 10:** Number of blades sensitivity analysis.

Z	6			8			10		
RPM	Δp	$\dot{W_{c}}$	η	Δp	$\dot{W_{c}}$	η	Δp	$\dot{W_{c}}$	η
12,870	17.751	37.36	0.4751	18.128	38.52	0.4706	18.116	39.15	0.4627
16,300	29.657	78.01	0.3802	30.320	79.81	0.3799	29.176	81.42	0.3583
18,500	37.869	111.23	0.3404	38.932	116.00	0.3356	38.170	117.26	0.3255

Units: Δp [mbar]; $\dot{W_{c}}$ [Watt].

**Table 11:** Impeller outlet width sensitivity analysis.

*b*_2_	4			3			2		
RPM	Δp	$\dot{W_{c}}$	η	Δp	$\dot{W_{c}}$	η	Δp	$\dot{W_{c}}$	η
12,870	18.12	39.15	0.4627	17.80	36.24	0.4912	15.40	31.35	0.4913
16,300	29.18	81.42	0.3583	27.98	72.32	0.3869	24.50	62.77	0.3904
18,500	38.17	117.26	0.3255	36.31	101.41	0.3581	30.77	90.28	0.3408

Units: Δp [mbar]; $\dot{W_{c}}$ [Watt].

**Table 12:** Main dimensions of the ORP impeller.

Parameter	Value	Units
$\beta_{1}$	25	°
$\beta_{2}$	15	°
$d_{1}$	23	mm
$d_{2}$	60	mm
$b_{1}$	10	mm
$b_{2}$	2.5	mm
*z*	6	-
*s*	1	mm

## Data Availability

Not applicable.

## Abbreviations

The following abbreviations are used in this manuscript:

CAD	Computer Aided Design
CFD	Computer Fluid Dynamics
DC	Direct Current
DoF	Degrees of Freedom
ESC	Electronic Speed Controller
LIDAR	Laser Imaging Detection and Ranging
Li-Po	Lithium-ion polymer
MRF	Multiple Reference Frame
NASA	National Aeronautics and Space Administration
ORP	Onboard Robot Prototype
ROMERIN	Modular Climbing Robot for Infrastructure Inspection
RPM	Revolutions per Minute
SST	Shear Stress Transport
TBP	Test Bench Prototype
